# Cost comparison between open radical cystectomy, laparoscopic radical cystectomy, and robot-assisted radical cystectomy for patients with bladder cancer: a systematic review of segmental costs

**DOI:** 10.1186/s12894-019-0533-x

**Published:** 2019-11-08

**Authors:** Yasuhiro Morii, Takahiro Osawa, Teppei Suzuki, Nobuo Shinohara, Toru Harabayashi, Tomoki Ishikawa, Takumi Tanikawa, Hiroko Yamashina, Katsuhiko Ogasawara

**Affiliations:** 1 Graduate school of Health Sciences, Hokkaido University, N12-W5, Kitaku, Sapporo, Hokkaido Japan; 2 Department of Renal and Genitourinary Surgery Graduate School of Medicine, Hokkaido University, N14, W5, KitaKu, Sapporo, Hokkaido Japan; 3 Hokkaido University of Education, Art, and Sports Business, Sapporo, Hokkaido Japan; 4 Faculty of Health Sciences, Hokkaido University, N12-W5, Kitaku, Sapporo, Hokkaido Japan; 5 Department of Urology, Hokkaido Cancer Center, 3-54, Kikusui 4-2, Shiroishiku, Sapporo, Hokkaido Japan; 6 Institute for Health Economics and Policy, No.11 Toyo-kaiji Bldg, 1-5-11, Nishi-Shimbashi,Minato-ku, Tokyo, Japan; 7 Faculty of Health Sciences, Hokkaido University of Science, 7-Jo 15-4-1 Maeda, Teine, Sapporo, Hokkaido Japan

## Abstract

**Background:**

Robot-assisted radical cystectomy is becoming a common treatment for bladder carcinoma. However, in comparison with open radical cystectomy, its cost-effectiveness has not been confirmed. Although few published reviews have compared total costs between the two surgical procedures, no study has compared segmental costs and explained their impact on total costs.

**Methods:**

A systematic review was conducted based on studies on the segmental costs of open, laparoscopic, and robot-assisted radical cystectomy using PubMed, Web of Science, and Cochrane Library databases to provide insight into cost-effective management methods for radical cystectomy. The segmental costs included operating, robot-related, complication, and length of stay costs. A sensitivity analysis was conducted to determine the impact of the annual number of cases on the per-case robot-related costs.

**Results:**

We identified two studies that compared open and laparoscopic surgeries and nine that compared open and robotic surgeries. Open radical cystectomy costs were higher than those of robotic surgeries in two retrospective single-institution studies, while robot-assisted radical cystectomy costs were higher in 1 retrospective single-institution study, 1 randomized controlled trial, and 4 large database studies. Operating costs were higher for robotic surgery, and accounted for 63.1–70.5% of the total robotic surgery cost. Sensitivity analysis revealed that robot-related costs were not a large proportion of total surgery costs in institutions with a large number of cases but accounted for a large proportion of total costs in centers with a small number of cases.

**Conclusions:**

The results show that robot-assisted radical cystectomy is more expensive than open radical cystectomy. The most effective methods to decrease costs associated with robotic surgery include a decrease in operating time and an increase in the number of cases. Further research is required on the cost-effectiveness of surgeries, including quality measures such as quality of life and quality-adjusted life years.

## Background

Radical cystectomy is a standard surgical technique for non-metastatic muscle-invasive bladder carcinoma [1]. Open radical cystectomy (ORC) has been the gold standard treatment method, while laparoscopic radical cystectomy (LRC) has also been used. The safety and efficacy of LRC have been well-documented [2]. Recently, robot-assisted radical cystectomy (RARC) has become increasingly common [3, 4]. The safety and efficacy of RARC compared to those of ORC have also been reported [4–6]. In addition, previous randomized controlled trials (RCTs) have reported no significant differences in 2-year progression-free survival rates [7] and quality of life (QOL) scores between ORC and RARC [8, 9]. Comparing the perioperative outcomes of ORC and RARC, Tang et al. [10] conducted a meta-analysis of several RCTs and reported significantly lower estimated blood loss (EBL), lower transfusion rates, longer operative times, and larger quantities of anesthesia used with RARC. Although surgical outcomes are important, cost-effectiveness is also of great significance if RARC is to be widely adopted [11]. In addition, as bladder carcinoma is reported to have the highest lifetime treatment costs per patient among all malignancies [12], the cost-effectiveness of bladder carcinoma treatments needs to be evaluated. Smith et al. conducted a cost analysis between ORC and RARC [11], and the results show a total cost advantage of $1630/case for RARC. In contrast, the cost-analysis by Bansal et al. showed a total cost advantage of approximately $1945/case for ORC [13]. Michels et al. [14] conducted a cost simulation of ORC and RARC and found that RARC costs €3365 more than ORC at 30 days. These studies indicated that operative time, length of stay (LOS), and the number of annual cases were key drivers of costs [14]. However, consensus regarding the most cost-effective surgical approach is yet to be reached. Although some reviews have been published on cost comparisons between surgical procedures [15, 16], they do not clarify the cost structure or focus on the total cost and not on segmental costs or cost-effective measures for robotic surgery. For hospitals, identification of the cost components that influence the total cost is crucial to make RARC more cost-effective. Therefore, we conducted a systematic review on the segmental costs of ORC, LRC, and RARC. This study aimed to provide cost-effectiveness data for ORC, LRC, and RARC and provide insights for the effective management of treatments and applicability of RARC for patients with bladder cancer. This study also aimed to clarify the current available knowledge to identify any gaps in order to promote future research.

## Methods

### Study selection and risk of bias assessment

This review was conducted according to the guidelines from the Preferred Reporting Items for Systematic Review and Meta-Analysis [17]. Two reviewers (Y.M. and T.O.) independently identified potentially relevant studies. The search was conducted using PubMed, Web of Science, and Cochrane Library databases on April 26, 2018. The search term was a combination of (bladder cancer) AND (radical cystectomy OR open radical cystectomy OR laparoscopic radical cystectomy OR robot-assisted radical cystectomy OR robotic radical cystectomy) AND (cost OR cost analysis OR cost-effectiveness OR cost utility analysis OR health technology assessment OR incremental cost effectiveness ratio OR ICER). The search terms were identified from “all texts.” Studies comparing costs for ORC to RARC or ORC to LRC in the form of full articles written in English were included. Simulation studies and studies with cost analysis not from a hospital perspective were excluded. When there was a difference of opinion regarding the inclusion of an article, it was resolved by other coauthors. To evaluate potential bias in included studies, “risk of bias” analysis of the included non-database studies was performed using the Cochrane Risk of Bias Tool [18].

### Data extraction and outcomes of interest

The reviewers independently extracted the following data (whenever available), including the first author, year of publication, country, study period, study design (whether the article was an RCT or a retrospective study), database used (in cases of database research), types of surgical procedure, number of patients who underwent ORC, LRC or RARC; type of urinary diversion (intracorporeal or extracorporeal), procedure for lymph node dissection, robot used for RARC, amortization period for the robot, and annual number of robotic cases. Perioperative outcomes were extracted from non-database studies, such as operative time, operating room occupancy time, EBL, blood transfusion rate, LOS, and complication rates. The extracted outcome measures (whenever data was available) included: types of cost, what the cost included, quality of life (QOL), quality-adjusted life years (QALY), and incremental cost effectiveness ratio (ICER). The data on perioperative cost was classified into four groups: operating, complication, total LOS, and robot-related costs. Operating costs included costs related to surgery (surgical equipment, personnel, operating room occupancy, and anesthesia). Complication costs included any cost related to perioperative complications within 90 days after surgery (including costs for complication treatments, readmission due to complication, and transfusion). Robot-related costs consisted of the initial robot purchase and annual maintenance fees. Per-case robot-related costs were also extracted. A sensitivity analysis on the per-case robot-related cost was conducted to analyze the effect of the number of cases on the total RARC costs. The costs were converted to US dollars using the currency exchange rate as of August 29, 2018. Next, the contribution of each cost segment to the total cost was calculated whenever possible. When there was incomplete or missing data essential for the systematic review, the reviewers attempted to contact the corresponding author of the article.

## Results

### Study selection

**Fig. 1:**
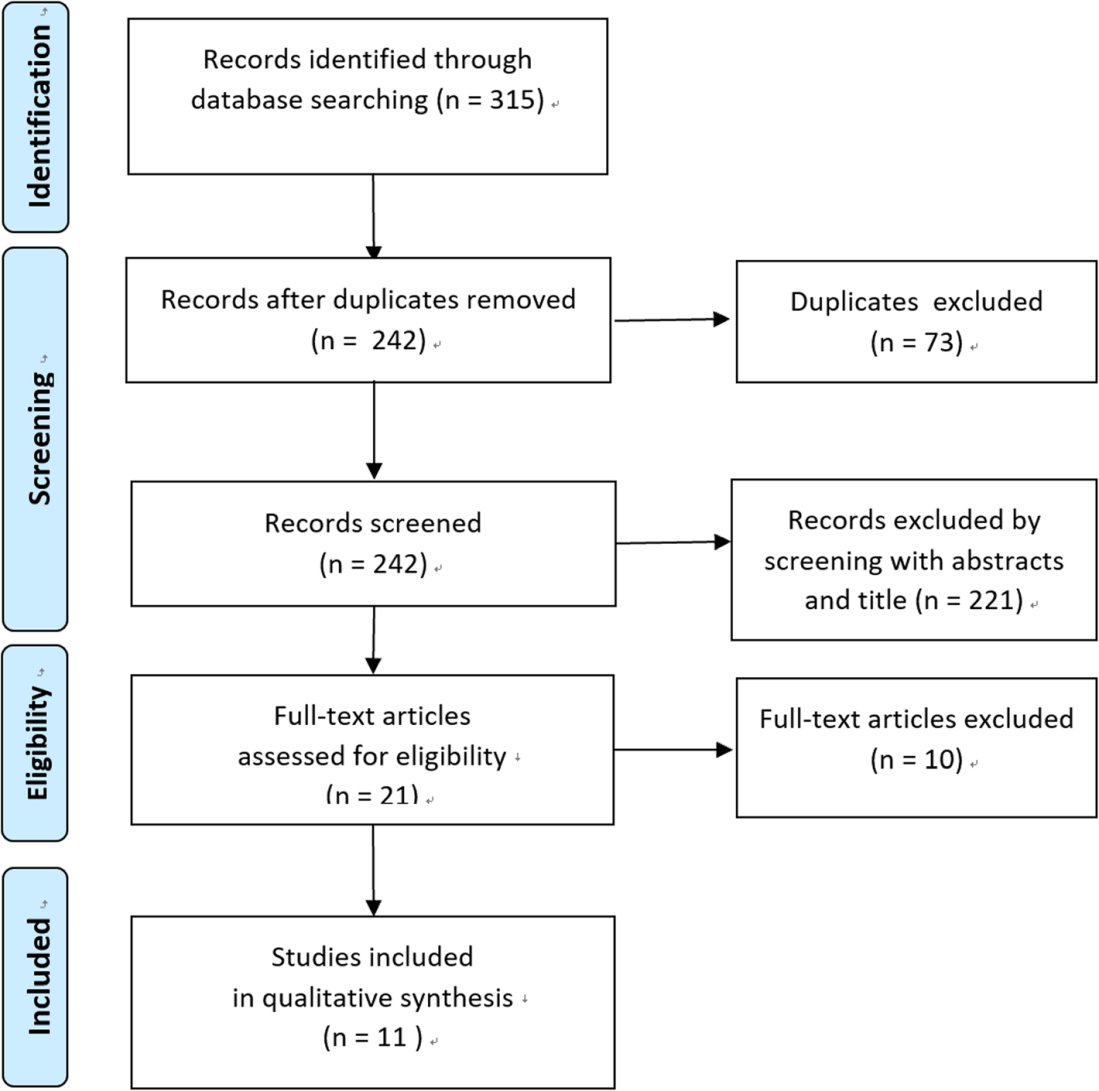
Flow chart of study selection. Flow chart of study selection. From the databases, 315 studies were identified. After removing duplicates and screening of titles, abstracts, and full texts, 11 studies were included for analysis

A flow chart of study selection is shown in Fig. 1. We initially identified 315 studies from PubMed, Web of Science, and Cochrane Library databases. Of the 315 studies, 11 were included in this study [11, 13, 19–27]. The characteristics of the included studies are listed in Table 1. Two studies compared ORC to LRC [19, 20], and nine compared ORC to RARC [11, 13, 21–27]. Of the studies that compared ORC to RARC, four were single-institutional retrospective studies [11, 13, 21, 22], one was a single-institutional RCT [23], and the remaining four were administrative large database studies [24–27]. The QOL was measured in one study [23] and no significant difference in QOL scores between ORC and RARC was found. No research was done using QALY or ICER. Intracorporeal urinary diversion was performed in the study by Bansal et al., while all other studies reported on extracorporeal urinary diversion. The rates of urinary diversion types performed are shown in Table 2.

**Table 1:** Characteristics of the included studies

Author	Country	Year	# of ORC case	# of LRC case	# of RARC case	Study Period	Study type (The name of databases used)
Bansal [13]	UK	2017	68		221	2011–2016	Single-institutional retrospective study
Bochner [23]	USA	2014	58		60	2010–2013	Single-institutional RCT
Martin [30]	USA	2011	14		19	2006-	Single-institutional retrospective study
Lee [22]	USA	2010	103		83	2002–2009	Single-institutional retrospective study
Smith [11]	USA	2010	20		20	2006-	Single-institutional retrospective study
Hermans [19]	Netherlands	2014	44	42		2005–2012	Single-institutional retrospective study
Zheng [20]	China	2012	65	45		2004–2011	Single-institutional retrospective study
Yu [26]	USA	2012	1444		224	2009	Administrative database study (Nationwide Inpatient Sample)
Leow [24]	USA	2014	34,672		2101	2004–2010	Administrative database study (Premier Perspective Darabase)
Hu [25]	USA	2016	7308		439	2002–2012	Administrative database study (Surveillance, Epidemiology, and End Results Program and Medicare linked data)
Monn [27]	USA	2014	25,986		3733	2009–2011	Administrative Database study (Nationwide Impatient Sample)

The characteristics of the 11 studies that compared ORC to LRC or ORC to RARC. The studies consisted of six single-institutional retrospective studies, a single-institutional RCT, and four database studies. The characteristics included information on authors, countries where the studies were performed, year of publication, number of cases (ORC, LRC or RARC), study periods, and study types. *RCT* Randomized controlled trial

**Table 2:** Per-case total costs and urinary diversion types performed

Author	Urinary diversion type (ORC) (% of total cases)	Urinary diversion type (RARC) (% of total cases)	Total cost (ORC)	Total cost (RARC)	Cost advantage for RARC
Bansal [13]	Ileal conduit (100%), Orthotopic neobladder (0%), Other (0%)	Ileal conduit (91.4%), Orthotopic neobladder (7.7%), Other (0.9%)	$13,512	$16,060	-$2548
Bochner [23]^a^	Ileal conduit (45%)	Ileal conduit (40%)	$16,648	$18,388	-$1740
	Orthotopic neobladder (55%)	Orthotopic neobladder (55%)	$15,311	$19,231	-$3920
Martin [21]	Ileal conduit (100%)	Ileal conduit (100%)	N.A. (for institutional reason)		
Lee [22] ^b^	Ileal conduit (50%)	Ileal conduit (57%)	$25,505	$20,659	$4846
	Continent cutaneous (22%)	Continent cutaneous (12%)	$22,697	$22,102	$595
	Orthotopic neobladder (28%)	Orthotopic neobladder (31%)	$20,719	$22,695	-$1976
Smith [11]	N.A.	N.A.	$16248^c^	$14608^c^	$1640
Yu [26]^b^	Ileal conduit (76.4%) Other (23.4%)	Ileal conduit (75.7%) Other (24.3%)	$28100^d^	$34303^d^	-$6203
Leow [24]	Ileal conduit (93.9%), Continent (6.1%)	Ileal conduit (91.5%), Orthotopic Continent (8.5%)	$26679^d^	$30974^d^	-$4295
Hu [25]	Incontinent (81.3%) Continent (4.1%)^e^	Incontinent (80.5%) Continent (5.0%)^e^	$32521^d^	$36121^d^	-$3600
Monn [27]	N.A.	N.A.	$25098^d^	$30272^d^	-$5174

Per-case total costs of ORC and RARC and the types and rates of urinary diversions performed in the included studies ^a^Patients with continent cutaneous tracts were not included in the analysis; ^b^no significant differences in the rates of urinary diversion types performed between ORC and RARC groups; ^c^surgeon fees were not included; and ^d^significant cost differences existed between ORC and RARC ^e^the rest is unknown

### Risk of bias evaluation

**Fig. 2:**
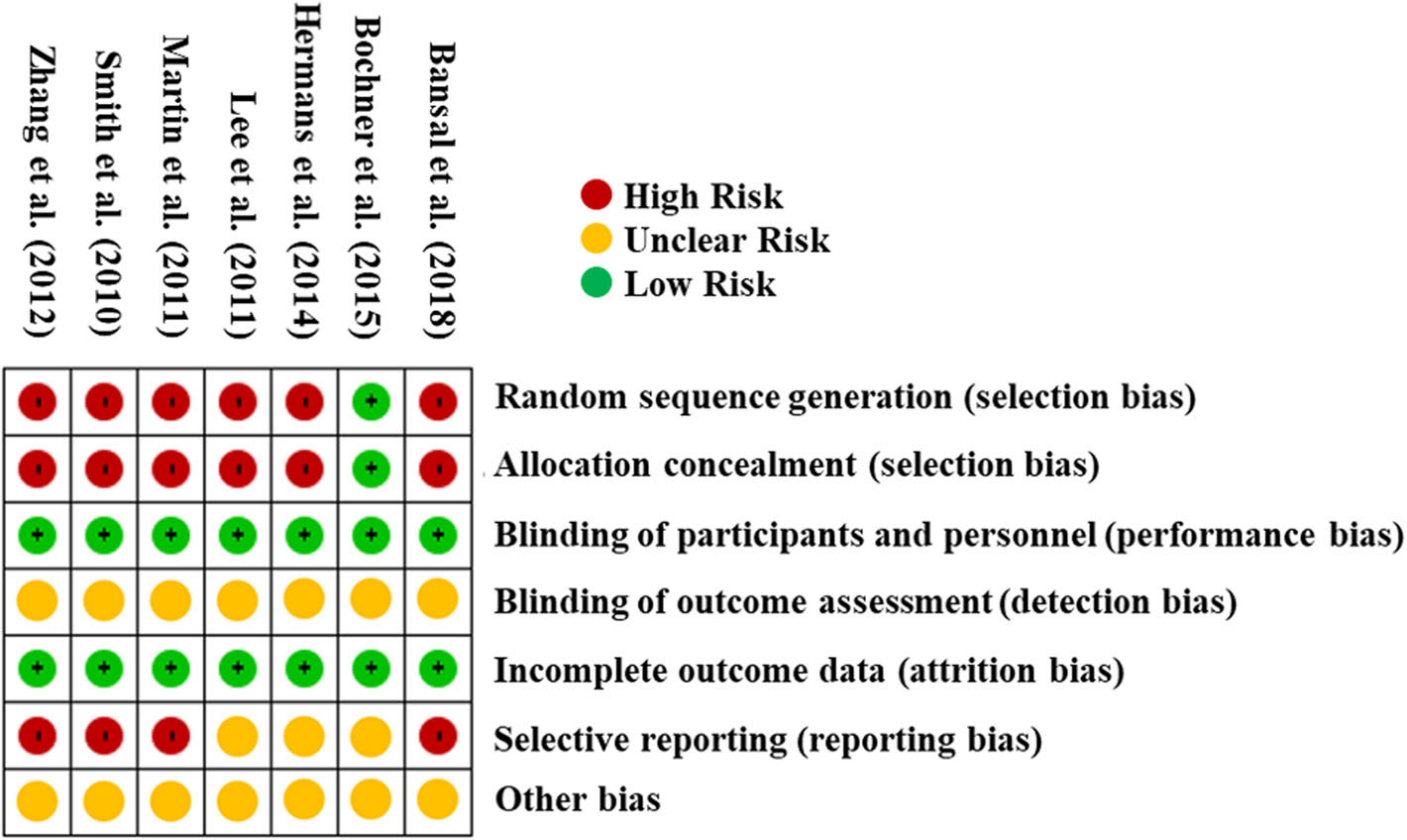
Result of risk of bias evaluation. The results of “risk of bias” assessment conducted using the Cochrane Risk of Bias Tool [18]

The results of the “risk of bias” evaluation are shown in Fig. 2. All studies but one were considered “High risk” in “Random sequence generation” and “Allocation concealment” as they were retrospective studies. Blinding of participants and personnel was considered “Low risk” because blinding cannot be achieved in operation theatre settings, and allocation does not affect outcomes. Four articles were considered “High risk” in “Selective reporting” because of missing primary outcomes or cost data. For example, Martin et al. did not publish the exact cost data in their article for proprietary reasons [21], and Smith et al. did not include surgeon fees in their analysis [11].

### ORC vs LRC

Two studies compared ORC with LRC [19, 20]. According to a study by Hermans, cost did not significantly differ between ORC and LRC [19]. The mean and median direct healthcare costs per patient (operating room occupation, disposable surgical equipment, blood transfusions, and hospital stay costs) were $21,177 and $19,941 in the LRC group, and $26,914 and $19,214 in the ORC group, respectively. Mean operating time was significantly shorter with ORC, resulting in a lower operating room occupation cost ($6273 for ORC, and $7740 for LRC). LRC was associated with significantly lower costs of packed cells, ($878 vs $175), nursing ($12,066 vs $8211), and intensive care ($5417 vs $1177). Zheng et al. analyzed the total costs and found that LRC was significantly more expensive in comparison to ORC ($9993 vs $8197) [20].

### ORC vs RARC

#### Total costs

**Fig. 3:**
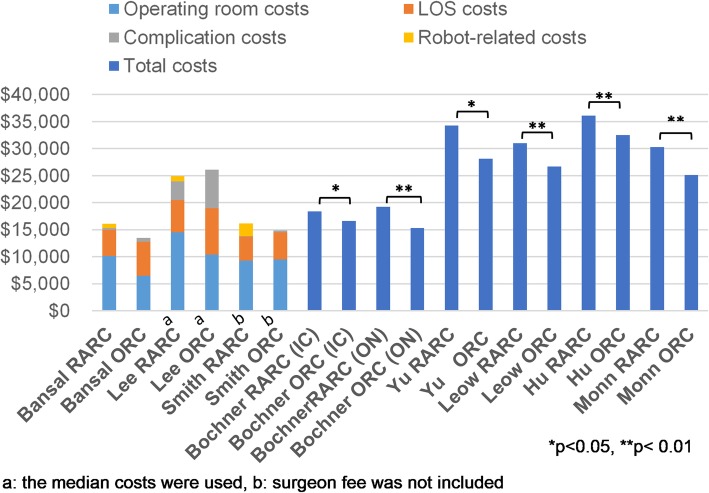
Summary of total costs and segmental costs for ORC and RARC. Summary of segmental and total costs in the included studies. Segmental costs are included whenever available; otherwise, total costs are shown. IC: Ileal conduit; ON: Orthotopic Neobladder

The information regarding per-case total costs is summarized in Table 2. Total costs and the segmental costs are shown in Fig. 3. A single-institutional retrospective study reported that ORC had a cost advantage of $2548 [13], while two single-institutional retrospective studies reported that RARC had a cost advantage [11, 21]. Martin et al. published that RARC was 38% less expensive than ORC although their research did not publish the exact cost data for proprietary reasons [21]. The results from one RCT and 4 database studies showed that RARC was $1740–$6203 more expensive [23–27]. Lee et al. subdivided their cohorts into radical cystectomy with ileal conduit, orthotopic neobladder, and cutaneous continent diversion subgroups, and compared the costs between ORC and RARC for these three subgroups [22]. In their research, RARC was less expensive than ORC for the ileal conduit, and cutaneous continent diversion subgroups; although, RARC was more expensive for the orthotopic neobladder subgroup.

#### Robot-related costs

**Fig. 4:**
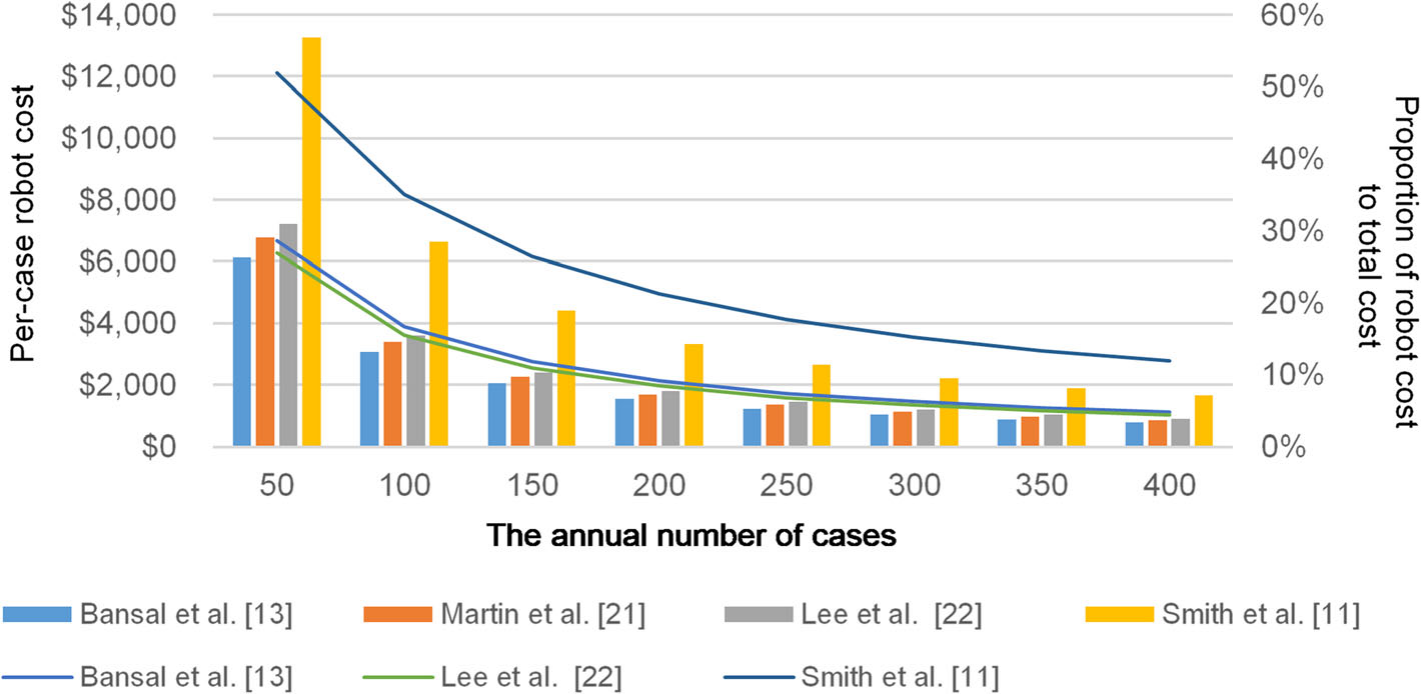
The effect of the annual number of cases on per-case robot-related costs. The per-case robot-related costs and rate of robot-related costs for the total RARC costs when the annual number of cases was changed from 50 to 400. Amortization periods are similar to those in Table 3. The per-case robot-related costs were calculated by amortizing the robot costs and distributing the costs to each case similar to the studies included in this review

The data on robot-related cost is presented in Table 3. In most studies, maintenance and purchase costs were not available. Robot purchase cost was shown only in one article (da Vinci© Surgical System: $1,650,000) [22]. Different amortization periods were used in the studies (5–10 years). The annual number of robotic-surgery cases varied from 288 to 400, and some included cases of other surgeries such as prostatectomies [11]. The per-case robot-related costs ranged from $766 to $2303 and were affected by amortization periods and the number of annual robot-surgery cases. The robot-related costs accounted for 4.8% of the total costs according to Bansal et al. [13], 4.8% of ileal conduit costs according to Lee et al. [21], and 15.8% according to Smith et al. [11]. Figure 4 shows the results of sensitivity analysis to clarify the effect of the annual number of cases on per-case robot-related costs. For example, when the annual number of cases was 50, the per-case robot-related cost would be $6128 of the total costs in the study by Bansal et al. (28.6% of the total RARC costs) [13], $6768 in the study by Martin et al. [21], $7220 in the study by Lee et al. (25.0–26.9%) [22], and $13,265 in the study by Smith et al. (51.9%) [11]. When the annual number of cases was 400, the per-case robot-related cost was $766 in the study by Bansal et al. (4.8% of the total RARC costs) [13], $846 in the study by Martin et al. [21], $903 in the study by Lee et al. (4.0–4.4%) [22], $1658 in the study by Smith et al. (11.9%) [11]. Per-case robot-related costs differed greatly depending on the annual number of cases.

**Table 3:** Robot-related costs

Author	Purchase cost	Amortization period	Annual maintenance cost	# of case per year	Total per-case cost
Bansal [13]	N.A.	10-year	$323/case	400	$766
Bochner [23]	N.A.	N.A.	N.A.	N.A.	N.A.
Martin [21]	N.A.	7-year	N.A.	300	$1128
Lee [22]	$1,650,000	7-year	$125,000 (347$/case)	361	$1000
Smith [11]	N.A.	5-year	N.A.	288 (including prostatectomy cases)	$2303

Robot-related cost data of the included studies. Robot-related costs included robot purchase costs and annual maintenance costs. The robot-related costs were calculated using the amortization periods and the annual number of cases in the institutions. The data on amortization periods and annual number of cases are also listed in this table

#### Complication costs

The data on perioperative complication costs are presented in Table 4. Complication rates were higher for ORC in two retrospective single-institutional studies [13, 22]. Although Bochner et al. did not publish complication costs, complication rates did not significantly differ between the two groups in their RCT. In addition, a database study did not show a significant difference in complication rates between ORC and RARC [23]. Complication costs were published in two single-institutional retrospective studies [13, 22], which reported that complication costs were higher in ORC. Moreover, although Martin et al. did not publish data on cost for proprietary reasons in their single-institutional retrospective study [21], the results indicated that the average complication cost associated with RARC was 60% less than that for ORC.

**Table 4:** Perioperative complication costs and proportion of the costs in the total costs

Author	Urinary diversion	Complication cost (ORC) (%)	Complication cost (RARC) (%)	Transfusion costs (ORC) (%)	Transfusion costs (RARC) (%)
Bansal [13]	Ileal conduit	$619 (5.1%)	$289 (2.3%)	$115 (0.9%)	$28 (0.2%)
Bochner [23]	Ileal conduit, Orthotopic neobladder	Included in the total cost		Included in the total cost	
Martin [21]	Ileal conduit	N.A. (for institutional reason)		N.A. (for institutional reason)	
Lee [22]	Ileal conduit	$7202 (28.2%)	$1624 (7.9%)	N.A	
	Orthotopic neobladder	$1663 (7.3%)	$1823 (8.2%)	N.A	
	Continent cutaneous	$2520 (12.2%)	$,911 (8.4%)	N.A	
	Total	$7103	$3482	N.A	
Smith [11]	N.A.	Not included in the analysis		$322 (2.0%)	$107 (0.7%)

Complication rates and costs, and the proportion of the total costs. The complication costs included complication treatment, readmission, and transfusion costs

Transfusion rates [13] and EBL [21, 23] were higher in ORC. Transfusion-associated costs were available in only two studies [11, 13], in which transfusion costs for ORC were more expensive than those of RARC ($115 vs. $28 and $322 vs. $107), and accounted for 0.2–2.0% of the total costs. Data on complication-associated costs based on the Clavien-Dindo grade were not available in any of the studies.

#### Operating costs

The data on operating costs is presented in Table 5. The mean operative time ranged from 228 to 420 min for ORC, and from 192 to 456 min for RARC. Operative time was longer for RARC, and operating costs were higher for RARC in all but one study [20]. Operating room occupancy time was not reported in the included studies. Operating costs accounted for 40.7–57.3% and 58.5–70.3% of the total costs of ORC and RARC, respectively. Lee et al. presented operating costs as median costs. Smith et al. did not include surgeon fees in their analysis. In the studies, no significant difference was found in the number of lymph nodes dissected. An RCT by Bochner et al. included patients who underwent standard or extended lymph node dissections, and the rates of patients who underwent extended dissections were matched between groups [22].

**Table 5:** Operating costs

Author	Urinary diversion	Operating cost (ORC) (%)	Operating cost (RARC) (%)	ORC mean operating time (min)	RARC mean operating time (min)
Bansal [13]	Ileal conduit, Orthotopic neobladder	$6464 (47.8%)	$10,140 (63.1%)	192^a^	265^a^
Bochner [23]	Ileal conduit, Orthotopic neobladder	included in total cost		330^b^	464^b^
Martin [21]	Ileal conduit	N.A. (for institutional reason)		320^a^	280^a^
Lee [22]^c^	Ileal conduit, Orthotopic neobladder, Continent cutaneous	$10,384 (40.7–50.1%)	$14,556 (64.1–70.5%)	420^d^	444
Smith [11]	N.A.	$9304 (57.3%)	$9527 (65.3%)	228^e^	246^e^

Operating costs, proportion of the costs to the total cost, and operative time of the included studies. The operating costs included operating room occupation costs, personnel fee, disposable equipment, and anesthesia. ^a^No statistical comparison; ^b^ significantly different between ORC and RARC; ^c^presented as median costs; ^d^surgeon fees not included; and ^e^occupancy time was reported

#### Length of stay cost

Data on LOS costs are presented in Table 6. The mean LOS ranged from 3.5–12.5 days and 3–10 days for ORC and RARC, respectively. The mean LOS did not differ in the RCT by Bochner et al. [23]. LOS costs were lower for RARC in all single-institutional studies. LOS costs accounted for approximately 30.7–46.7% and 26.0–30.2% of the total costs for ORC and RARC, respectively. Lee et al. used median costs [22]. Bansal et al. used NHS reference costs to estimate the costs for excess bed days [13].

**Table 6:** Length of Stay costs

Author	Urinary diversion	Length of stay cost (ORC)	Length of stay cost (RARC)	Mean length of stay (ORC) (days)	Mean length of stay (RARC) (days)
Bansal [13]	Ileal conduit, Orthotopic neobladder	$6314 (46.7%)	$4836 (30.1%)	12.5	8.8
Bochner [23]	Ileal conduit, Orthotopic neobladder	N.A. (included in total cost)		8	8
Martin [21]	Ileal conduit	N.A. (included in total cost)		10	5
Lee [22]^b^	Ileal conduit, Orthotopic neobladder, Continent cuteneous	$8592 (33.7–41.5%)	$5907 (26.0–28.6%)	8^a^	5.5^a^
Smith [11]	N.A.	$4982 (30.7%)	$4418 (30.2%)	5.3^b^	4.7^b^

LOS and related costs, and the proportion of the total costs in the included studies. ^a^Significantly different between ORC and RARC; ^b^costs are presented as median costs

## Discussion

Only one of the included single-institutional studies comparing ORC and RARC was an RCT while the other four were retrospective studies. In addition, the “risk of bias” evaluation showed that some studies could have “High risk” of “Selective reporting” as these studies did not report parts of the primary outcomes. Therefore, although these studies are important due to the lack of large studies comparing surgical procedures, more evidence from high quality studies (e.g. RCT) is required. Nevertheless, the results from single-institutional studies can provide insights on cost structures through interpretation with the included large database studies, which reflect the general cost trends.

Two single-institutional retrospective studies reported total cost advantages for RARC over ORC [21, 22]; another single-institutional retrospective study, one RCT, and four database studies showed total cost advantages for ORC [13, 22–26]. Considering the quality of the studies, the results indicated that, in general, RARC was more likely to be expensive. Michels et al. conducted a cost simulation of ORC and RARC using data from a literature review and reported that RARC was more expensive [14], similar to our study result. Leow et al. reported that the cost advantage in ORC was due to the additional costs of purchasing and maintaining robots and longer operative times for RARC [24, 28]. However, on the other hand, Martin et al. reported that the cost advantage in RARC was due to the lower complication rates [21]. These conflicting findings indicate that RARC cost-effectiveness was institution-dependent. Leow et al. reported that the surgical approach (robot-assisted vs open) was neither a major factor on cost variations nor associated with high costs [28]. Therefore, focusing on the segmental costs, such as operating costs and robot costs, is necessary to figure out how each aspect contributes to the total cost. The results of segmental costs from this study are of great importance for improving cost-effectiveness of RARC compared to that of ORC, from the hospital’s perspective. Per-case robot costs were calculated by amortizing the robot-related costs and dividing the costs by the annual number of cases in the subject hospitals, including cases of other surgeries such as prostatectomy. According to a previous study, robot equipment costs were attributed to higher costs in RARC [21]. Although robots were an expensive initial investment for an institution, the per-case robot-related costs accounted for only 4.8–15.8% of the total costs (Table 3) [11, 13, 22] because the included single-institutional studies were conducted in high volume hospitals (288–400 cases per year). On the other hand, sensitivity analysis (Fig. 4) revealed that per-case robot-related costs differed greatly depending on the annual number of cases. High-volume centers were more likely to have lower per-case robot-related costs [24], while these costs tended to be higher in low-volume institutions.

The complication costs were higher for ORC in three single-institutional retrospective studies [13, 21, 22], accounting for 1.9% [13] and 16.7% [22] of the total costs. On the other hand, Bochner et al. showed in an RCT that there was no significant difference in perioperative complication rates between ORC and RARC, although the research did not report segmental costs for complications [23]. A previous meta-analysis of four RCTs by Tang et al. revealed no significant differences between groups in the occurrence rates of patients with Clavien-Dindo grade 2–5 or 3–5 [10]; therefore, complication costs may not differ between groups. Although complication costs differ with Clavien-Dindo grades, none of the included studies showed complication costs according to the Clavien grade at 90 days, which is a standard method of reporting postoperative complications. Further studies on complication costs are required with a high-level of evidence. Additionally, it is recommended that future studies focus on complication costs according to the Clavien-Dindo grade.

Complication costs can differ among countries. A multi-institutional study by Osawa et al. reported that causes of complications differed between the USA and Japan [30]. International comparisons of complication costs also need to be conducted carefully.

Differences in transfusion rates between ORC and RARC have been reported in various studies [10]. However, our results showed that even though transfusion rates were clinically essential, the difference did not largely affect the total costs.

The operating costs of RARC were higher in all studies due to longer operative times, which was similar to previous reports [10]. Operating costs accounted for approximately 63.1–70.5% of the total RARC cost (Table 5 and Fig. 3). Most of the operating time costs were attributed to operating room occupation and surgeon fees which are dependent on the operative time. Therefore, if an institution succeeds in shortening the operating time, it would effectively reduce the total cost. Operative time has been reported to decrease significantly with increased surgeon experience [31] and hospital volume [29]. Leow et al. reported that although total costs were significantly higher in the RARC group, the difference did not exist between high-volume surgeons (≧7 cases per year in their study) and hospitals (≧19 cases per year) [24]. Large institutions can benefit from shorter operative times, lower complication rates, cheaper per-case robot costs, and therefore, achieve more cost-effective RARC. Patient centralization to high-volume centers has been suggested as an effective way for cost-effective RARC surgeries [24], which is supported by our results. However, further research is required to reveal the relationship between a surgeons’ learning curve and cost-effectiveness of RARC.

Most studies were analyzed using operative time [13, 21, 22], except which used operating room occupancy time (utilization time) [11]. Operating rooms are essential for hospital profitability and thus, longer operating room occupation is associated with higher costs. For accurate cost estimations, it is recommended that these two parameters should be recorded and analyzed.

Urinary diversion types chosen can also influence the total cost. The results by Lee et al. showed that RARC was more cost-efficient for ileal conduit ($4846), while the cost benefit diminished for cutaneous continent diversion ($609), and was absent for orthotopic neobladder (−$1966; Table 2) [22]. This is one of the few studies that compare ORC with RARC by urinary diversion types. Current evidence on the impact of urinary diversion types on the costs is inadequate.

Recently, intracorporeal urinary diversions have become increasingly common [32]. Only one article included in this study was conducted with an intracorporeal urinary diversion [13]. Further studies are necessary to evaluate whether an intracorporeal or extracorporeal urinary diversion can influence operative time, and subsequently, the total costs.

Lymph node dissections differ depending on the surgeon and institution. Bochner et al. included patients who had undergone standard or extended lymph node dissections. In their RCT, the rates of patients who underwent extended dissections were matched between the ORC and RARC groups [23]. Lymph node dissections should be included when comparing ORC and RARC costs because extended dissections can lead to longer operating times and higher costs.

Three retrospective studies reported lower LOS costs for RARC. LOS costs also accounted for a large proportion of the total costs, following operating costs. However, Tang et al. conducted a meta-analysis of four RCTs and found no significant difference in LOS for ORC and RARC [10]. Of the studies included in this study, an RCT by Bochner et al. reported no significant difference of LOS between ORC and RARC. A database study by Leow et al. reported that while LOS differed significantly between ORC and RARC, LOS-related costs did not differ since most of the costs were due to surgery and intensive care unit admission [24]. Therefore, it is likely that LOS costs do not necessarily have a large impact on the differences between ORC and RARC costs.

LOS differs greatly between countries. Sugihara et al. compared the LOS of radical cystectomy patients in Japan and the USA and reported shorter durations in the USA (8 [7–11] days vs 32 [21–44] days) [33]. The effects of LOS changes should be considered with each country’s healthcare system [34].

Few studies mention the perioperative protocols used in their studies (e.g. ERAS protocols), making it difficult to compare results between studies. Nabhani et al. reported that using the ERAS protocol led to a cost saving of $4488/procedure [35]. Future studies are expected to be conducted under standardized protocols for more generalizable results.

One study measured QOL for ORC and RARC patients and found no significant difference between groups. This finding supports the results of Messer et al. and Khan et al. [8, 9]. None of the included studies analyzed cost-effectiveness using quality measurements, such as QALY; therefore, it is recommended that future studies should focus on cost-effectiveness.

Comparison of ORC and LRC was done in only two studies that had differing conclusions on which procedure was more expensive. Further studies are required to clarify and confirm which procedure is more cost-effective.

This is the first systematic review on the segmental costs of radical cystectomy to identify which cost segments impact the total cost. Therefore, the results of this research are significant to understand cost structure and consider how RARC can be cost-effective. However, this research has several limitations. First, medical systems differ to certain extent between countries [34]. Therefore, the results should be interpreted along with each country’s healthcare system. Correcting the segmental costs with references (e.g. NHS reference costs) will enable cost differences between institutions to be partially. Second, although clinical practice such as the use of surgical equipment could differ among institutions and surgeons, the information (e.g. number or quantity of equipment used) was not explained in detail. However, we included the database studies [24–27], which allows for some generalizability of the surgeons and institutions. Finally, the study periods were up to 90 days postoperatively. Bladder carcinomas have the highest lifetime treatment cost per patient out of all malignancies [12]. Therefore, future research on the lifetime costs would be valuable.

## Conclusion

In this study, we systematically reviewed studies that compared costs of ORC, LRC, and RARC and segmented the costs into four groups to provide useful data for administrative purposes. The results revealed RARC to be more expensive. The results from the segmented costs indicated that RARC operating costs were higher and accounted for the largest proportion of total RARC costs. Sensitivity analysis revealed that the annual number of cases largely affected the per-case robot costs, and subsequently affected the total costs. Therefore, to make RARC cost-effective, a short operative time and high number of cases would be the most efficient method. Further studies focusing on complication costs with a high level of evidence is required. Data from this research can be used to make RARC more cost-effective than ORC. Future studies need to focus on the cost-effectiveness of ORC and RARC by using quality measurements, such as QALY; standardizing the methods of complication costs analyses (e.g. per Clavien-Dindo grade), and adopting standardized perioperative protocols, such as ERAS.

## Data Availability

All data generated or analyzed during this study are included in this published article.

## Abbreviations

EBL: Estimated blood loss
ICER: Incremental Cost Effectiveness Ratio
LOS: Length of stay
LRC: Laparoscopic radical cystectomy
ORC: Open radical cystectomy
QALY: Quality-adjusted life years
RARC: Robot assisted radical cystectomy
RCT: Randomized controlled trial
